# Mindfulness-Based Stress Reduction Benefits Psychological Well-Being, Sleep Quality, and Athletic Performance in Female Collegiate Rowers

**DOI:** 10.3389/fpsyg.2020.572980

**Published:** 2020-09-18

**Authors:** Bethany J. Jones, Sukhmanjit Kaur, Michele Miller, Rebecca M. C. Spencer

**Affiliations:** ^1^Department of Psychological and Brain Sciences, University of Massachusetts, Amherst, MA, United States; ^2^Neuroscience and Behavior Program, University of Massachusetts, Amherst, MA, United States; ^3^Commonwealth Honors College, University of Massachusetts, Amherst, MA, United States; ^4^Amherst Mindfulness, Amherst, MA, United States

**Keywords:** mindfulness training, collegiate athletics, endurance, rowing, actigraphy, coping skills

## Abstract

Factors such as psychological well-being, sleep quality, and athletic coping skills can influence athletic performance. Mindfulness-based interventions, including mindfulness-based stress reduction (MBSR), have been shown to benefit these factors, suggesting they may, at least indirectly, benefit athletic performance. Moreover, while mindfulness training has been linked to better accuracy in some high-precision sports, whether it can improve non-precision elements of athletic performance is unclear. The objective of this study was to investigate the influence of MBSR on psychological well-being, sleep, athletic coping skills, and rowing performance in collegiate rowers in a controlled experimental design. Members of a Division I NCAA Women’s Rowing team completed either an 8-week MBSR course along with their regular athletic training program (Intervention group) or the athletic training program alone (Control group). Measurements of interest were taken at baseline and again either during or shortly following the intervention. In contrast to the Control group, the Intervention group showed improvements in psychological well-being, subjective and objective sleep quality, athletic coping skills, and rowing performance as measured by a 6,000-m ergometer test. Improvements in athletic coping skills, psychological well-being, and subjective sleep quality were all correlated with increases in mindfulness in the Intervention group. These results suggest that mindfulness training may benefit non-precision aspects of athletic performance. Incorporating mindfulness training into athletic training programs may benefit quality of life and performance in student athletes.

## Introduction

Collegiate student-athletes face a wide array of stressors, ranging from those related to athletic performance and academics to adjusting to life away from home. Too much stress can negatively influence psychological well-being which, in turn, can negatively impact athletic performance (Reardon et al., 2019). Extensive stress can also impair sleep, another effect that may impede optimal performance. Both mental health symptoms and sleep problems are prevalent in elite athletes, and the National Collegiate Athletic Association as well as the International Olympic Committee have emphasized the need for improved sleep to benefit health and performance in this population (Kroshus et al., 2019; Reardon et al., 2019). Mindfulness-based stress reduction (MBSR) is a cognitive training program that can improve psychological well-being and sleep quality (Kabat-Zinn, 1982; Grossman et al., 2004; Cincotta et al., 2011; Li et al., 2018; Rusch et al., 2019), and thus may indirectly benefit athletic performance.

Psychological well-being influences athletic performance. Competition anxiety, trait anxiety, and fear of negative evaluation hinder performance. In both competition and training, athletes with lower levels of anxiety perform better than those with higher levels of anxiety (Hill et al., 2011; Castro-Sánchez et al., 2018). Under high-stress situations, athletes with fears of negative evaluation demonstrate increased competition anxiety and decreased performance (Hill et al., 2011; Geukes et al., 2017). On the other hand, mental toughness or the ability to remain “determined, focused, confident, and in control under pressure” (Jones, 2002) mediates the ability of athletes to excel under high-stress situations (Nicholls et al., 2015).

Adequate sleep is also essential for optimal athletic performance. For example, acute sleep deprivation has been found to impair endurance performance observed in runners (Oliver et al., 2009; Skein et al., 2011). In another study, collegiate basketball players were granted a 2-h sleep extension for 5–7 weeks. Follow-up measures showed increased sleep time as well as significant improvements in sprint time and shooting accuracy compared to baseline performance (Mah et al., 2011). The roles of sleep in muscle recovery, psychological well-being, reaction time, and accuracy may all support athletic performance (Watson, 2017). Despite the demonstrated importance of sleep for performance, elite and collegiate athletes often exhibit poor sleep quality and inadequate sleep duration (Leeder et al., 2012; Mah et al., 2018; Brauer et al., 2019; Kroshus et al., 2019; Reardon et al., 2019).

Mindfulness is a state of nonjudgmental awareness and acceptance of the present moment, including inner thoughts and emotions. MBSR is a standardized training program in mindfulness that has been shown to reduce stress, anxiety, depression, and pain (Kabat-Zinn, 1982; Grossman et al., 2004). MBSR also leads to improvements in sleep. In sleep-disturbed populations, mindfulness-based interventions improve sleep quality compared to placebo interventions (Rusch et al., 2019). In healthy adults, MBSR led to self-reported decreases in pre-sleep cognitive arousal and increases in sleep quality (Cincotta et al., 2011). Similarly, in athletes, brief mindfulness meditation prior to sleep led to reduced pre-sleep arousal and improved overall sleep quality (Li et al., 2018).

Mindfulness may benefit both sport-related coping skills and athletic performance. Higher trait mindfulness is associated with reduced competition anxiety, higher self-efficacy and sport confidence, and subjectively better ability to perform (Pineau et al., 2014; Röthlin et al., 2016). Rumination and emotion regulation have been shown to mediate the relationship between trait mindfulness and sport-specific coping skills in elite athletes (Josefsson et al., 2017). In line with these findings, mindfulness training was associated with decreased perceived stress and increased athletic coping skills in collegiate women basketball players (Vidic et al., 2017). Fewer studies have measured the effects of mindfulness on objective athletic performance. While there is evidence that mindfulness-based interventions benefit accuracy in precision sports such as dart throwing and shooting (Solberg et al., 1996; John et al., 2011; Zhang et al., 2016), it is unclear whether a benefit would extend to non-precision performance elements such as speed and endurance (Bühlmayer et al., 2017).

The goal of the current study was to investigate the effect of MBSR on psychological well-being, sleep quality, athletic coping skills, and objective athletic performance (rowing completion time) in collegiate rowers in a controlled experimental paradigm. Given past findings, we hypothesized that MBSR would lead to improvements in well-being, sleep quality, and coping skills, and that these improvements would be related to increased mindfulness. We reasoned that benefits in these variables would translate to better objective athletic performance as well.

## Materials and Methods

### Participants

All members of a Division I NCAA Women’s Rowing team who met inclusion criteria were invited to participate in this study. Inclusion criteria were having normal or corrected-to-normal vision and no history of neurological disease, sleep disorders, head injury, or use of medications known to affect sleep or cognitive function. Twenty-seven members with an age range of 18–23 years accepted this invitation. This sample size is consistent with those used in similar studies (e.g., Solberg et al., 1996; Vidic et al., 2017). All participants were compensated with payment. Experimental procedures were approved by the University of Massachusetts, Amherst Institutional Review Board, and written informed consent was obtained before the experiment.

### Rowing Apparatus

A Concept II ergometer was used to assess rowing-related performance. The ergometer simulates the motion and pressure of rowing on the water. A screen is attached to each machine and allows the rower to view information such as time elapsed, projected time to completion, distance rowed, stroke rate, and average time to row 500 m. This indoor machine provides a measure of athletic performance while controlling for environmental factors (e.g., wind, current, temperature, etc.) that could influence outcomes if athletes were tested on the water.

### Questionnaires

#### Athletic Coping Skills Inventory 28

This inventory consists of 28 items measured on a 4-point Likert scale rated from 0 (almost never) to 4 (almost always; Smith et al., 1995). The seven subscales, (1) Coping with Adversity, (2) Coachability, (3) Concentration, (4) Confidence and Achievement Motivation, (5) Goal Setting and Mental Preparation, (6) Peaking under Pressure, and (7) Freedom from Worry, combine to yield an overall, multifaceted-psychological coping score ranging from 0 to 84. Higher scores reflect increased ability to cope.

#### Beck Anxiety Inventory

This 21-item self-report questionnaire assesses anxiety over the past month (Beck et al., 1988). Items are rated on a 4-point Likert scale ranging from 0 (not at all) to 3 (severely – it bothered me a lot), with the total possible score ranging from 0 to 63. Higher scores reflect more severe anxiety.

#### Beck Depression Inventory II

This 21-item self-report questionnaire assesses depressive symptoms over the past 2 weeks (Beck et al., 1996). Items are rated on a 4-point Likert scale ranging from 0 to 3, with the total possible score ranging from 0 to 63. Higher scores reflect more severe depressive symptoms.

#### Epworth Sleepiness Scale

This eight-item questionnaire assesses daytime sleepiness by asking respondents to rate their likelihood of dozing off or falling asleep during different daytime activities (Johns, 1991). Items are rated on a 4-point Likert scale (0–3), with the total possible score ranging from 0 to 24. Higher scores reflect greater daytime sleepiness.

#### Five Facet Mindfulness Questionnaire

This 39-item self-report inventory assesses mindfulness tendency *via* five subscales: observing, describing, acting with awareness, non-judging of inner experience, and non-reactivity to inner experience (Baer et al., 2006). Items are rated on a 5-point Likert scale ranging from 1 (never or very rarely true) to 5 (very often or always true), with the total possible score ranging from 39 to 195. Higher scores reflect greater mindfulness tendencies.

#### Perceived Stress Scale

This 10-item self-report questionnaire assesses general stress levels over the past month (Cohen, 1988). Items are rated on a 5-point Likert scale ranging from 0 (never) to 4 (very often), with the total possible score ranging from 0 to 40. Higher scores reflect greater perceived levels of stress.

#### Pittsburg Sleep Quality Index

This self-report questionnaire assesses general sleep quality over the past month by querying seven domains: subjective sleep quality, sleep latency, sleep duration, habitual sleep efficiency (SE), sleep disturbances, use of sleep medication, and daytime dysfunction (Buysse et al., 1989). The total possible score ranges from 0 to 21, with higher scores reflecting poorer sleep quality.

#### Ruminative Responses Scale

This 22-item self-report questionnaire assesses rumination tendency (Nolen-Hoeksema and Morrow, 1991). Items are rated on a 4-point Likert scale ranging from 1 (almost never) to 4 (almost always), with a total possible score ranging from 22 to 88. Higher scores reflect greater rumination tendency.

#### Scales of Psychological Well-Being

This 42-item self-report questionnaire assesses six domains of psychological well-being: autonomy, environmental mastery, personal growth, positive relations, purpose in life, and self-acceptance (42-item version adapted from Ryff, 1989). Items are rated on a 6-point Likert scale ranging from 1 to 6, with the total possible score ranging from 42 to 252. Higher scores indicate more positive psychological well-being.

### Procedure

**Figure 1:**
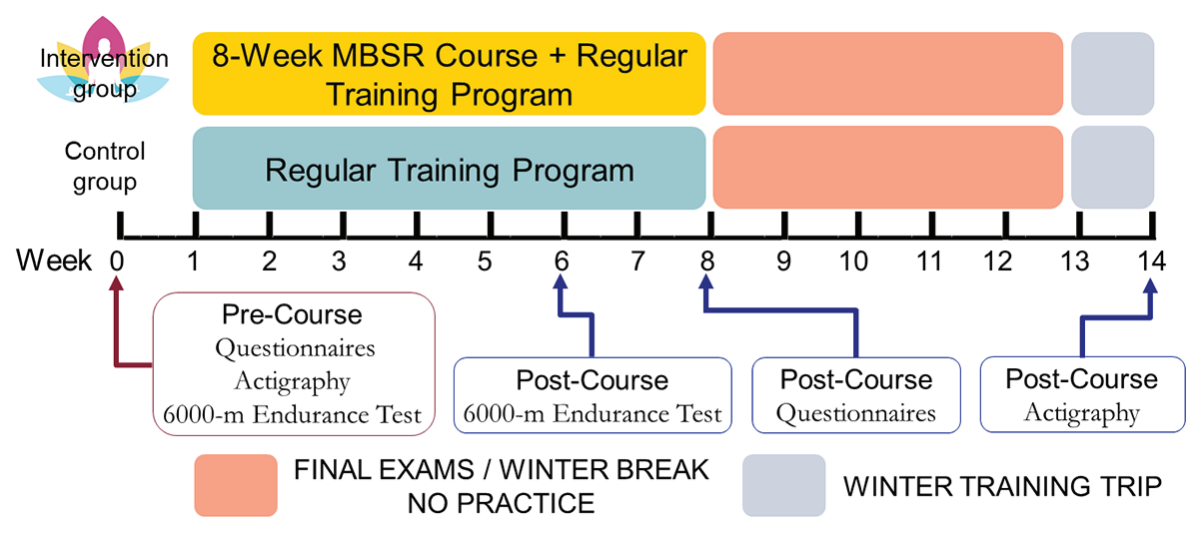
Experimental timeline and procedure. Participants either completed an 8-week mindfulness-based stress reduction (MBSR) course along with their regular athletic training program (Intervention group) or the athletic training program alone (Control group). Questionnaires, actigraphy, and an ergometer test were administered once at baseline and once again either during or shortly following the intervention.

Participants were quasi-randomly assigned to either the Intervention group (*n* = 15) or the Control group (*n* = 12) based on their availability during the scheduled MBSR class time. One week prior to the first session of the MBSR course, both groups completed a series of questionnaires regarding stress and emotional well-being, sleep, and athletic coping skills; 3–7 days of actigraphy; and a 6,000-m ergometer test conducted as part of the rowing team’s training regimen (Figure 1). After 6 weeks of the MBSR course, participants completed another 6,000-m ergometer test (originally, the MBSR course was scheduled to be completed prior to this second test. However, unforeseen adjustments to the schedule resulted in its occurrence during week 6). After finishing all 8 weeks of the MBSR course, participants completed a second round of questionnaires. Post-course actigraphy was collected at week 14 during the team’s winter training trip in order to match the consistent morning and afternoon practice schedules, which were in effect at the pre-course time-point. This timing avoided data collection during final exams and winter holidays, when students had varying schedules and a break in their athletic training.

Throughout the study, all athletes on the team followed the same training program which was developed by the coaches independent of this study. The coaches were informed of the study, but not of the predictions or group assignments to prevent any inadvertent discrepancies in coaching.

### MBSR Course

The course consisted of eight group classes that occurred over a 9-week period (one per week except during the holiday break). Each class session lasted 75 min and was led by a professional instructor. The class session duration was shortened from the standard 150 min to promote high attendance and more time for self-practice. Additionally, it was not feasible to include a day-long silent retreat in this adaptation due to the weekend practice schedule of the rowing team. The class was designed to facilitate mindfulness *via* guided meditations and discussions (Kabat-Zinn, 1982). All participants attended at least seven of the eight class sessions. In-class instruction was supplemented with handouts, workbooks, and audio recordings for in-home practice. Furthermore, participants were asked to engage in a minimum of 10 min of mindfulness practice daily on their own prior to sleeping.

### Actigraphy

A wrist-worn accelerometer (Actiwatch Spectrum Plus, Philips Respironics, Inc.) with a sampling rate of 32 Hz and sensitivity <0.01 g was used to objectively and reliably estimate overnight sleep (Sadeh et al., 1994). Activity was binned in 15-s epochs, and sleep was scored using Actiware software, according to the Society of Behavioral Sleep Medicine guidelines (Ancoli-Israel et al., 2015). Sleep onset and offset were identified manually in each record. Sleep onset was defined as the start of the first 3 consecutive min of sleep, and sleep offset was defined as the end of the last 5 consecutive min of sleep. Participants were asked to record their bedtime in a sleep diary and by pressing an event marker on the watch. However, due to poor compliance with these requests, time in bed and hence sleep onset latency could not be calculated. Thus, outcome variables were total sleep time (TST), wake after sleep onset (WASO), and adjusted SE, which were calculated as the percent of time between sleep onset and sleep offset that was spent asleep.

Participants with fewer than three nights of usable data at the pre-intervention and/or post-intervention time point were excluded from actigraphy analyses (MBSR group: *n* = 4; Control group: *n* = 4). Due to technical difficulty, data could not be retrieved from two additional participants (one from each group). Finally, one additional participant in the MBSR group was not available at the post-intervention time point used for actigraphy. Thus, actigraphy analyses are based on nine participants in the MBSR group and seven participants in the Control group.

### Data Analysis

Since we used multiple questionnaires [Beck Depression Inventory II (BDI-II), Beck Anxiety Inventory (BAI), Perceived Stress Scale (PSS), Ruminative Responses Scale (RRS), and Scales of Psychological Well-Being (SPWB)] to assess emotional and psychological well-being, many of which measure overlapping/co-linear constructs (e.g., stress, anxiety, and rumination), we combined scores on these questionnaires to create a composite measure of well-being. First, raw scores were converted to percentage scores by dividing them by the maximum possible score of the respective questionnaire. Next, because the Scales of Psychological Well-Being was the only questionnaire where higher scores reflect more positive well-being (in contrast to the others where higher scores reflect poorer well-being), its percentage scores were multiplied by −1 in order to make the interpretation of directionality consistent across all questionnaires. Finally, the percentage scores for these five questionnaires were summed for each individual at each time point. Thus, the total possible composite score ranges from −100 to 400, with lower scores reflecting better (more positive) well-being.

Statistical analyses were conducted in SPSS. Student’s independent-samples *t*-tests were used to investigate baseline differences between groups. Paired-samples *t*-tests were used to investigate changes in variables over time in the two groups. The Bonferroni correction was applied to correct for multiple comparisons (significance level set to *p* < 0.05/2 = *p* < 0.025). Pearson’s *r* was used to assess bivariate linear relationships. Multiple linear regressions were used to investigate mediation effects. One participant did not fill out all of the questionnaires at each time point, and thus was not included in the relevant analyses. Likewise, three participants did not complete the ergometer test at both time points due to injury or illness and were thus excluded from the ergometer analyses. Exclusions from actigraphy analyses are detailed above in the previous section. See Table 1 for final sample sizes in each measure.

**Table 1:** Baseline scores.

	MBSR			Control			
	*N*	*M*	*SEM*	*N*	*M*	*SEM*	*p*
*Mindfulness*							
FFMQ	15	118.60	5.59	10	119.10	6.64	0.955
*Emotional well-being*							
BDI-II	15	13.60	2.45	11	14.73	2.59	0.758
BAI	15	11.07	2.12	11	14.00	3.32	0.444
PSS	15	22.00	1.62	11	22.55	2.00	0.832
RRS	15	47.73	4.25	11	44.82	4.96	0.659
SPWB	14	174.86	8.67	11	167.32	7.95	0.538
Well-being composite	14	78.90	17.01	11	86.50	18.45	0.766
*Subjective sleep measures*							
ESS	15	9.73	1.45	11	11.27	1.29	0.455
PSQI	15	7.00	0.70	11	6.55	1.19	0.729
*Objective sleep measures*							
TST (min)	9	425.78	12.17	7	416.18	13.52	0.607
SE (%)	9	91.95	0.87	7	92.74	0.82	0.535
WASO (min)	9	37.82	4.41	7	32.32	3.30	0.360
*Athletic measures*							
ACSI-28	15	46.80	2.46	11	45.09	1.94	0.612
Ergometer time (min)	13	24.97	0.29	11	25.00	0.26	0.945

MBSR, mindfulness-based stress reduction; M, mean; SEM, standard error of the mean; FFMQ, Five Facet Mindfulness Questionnaire; BDI-II, Beck Depression Inventory II; BAI, Beck Anxiety Inventory; PSS, Perceived Stress Scale; RRS, Ruminative Responses Scale; SPWB, Scales of Psychological Well-Being; ESS, Epworth Sleepiness Scale; PSQI, Pittsburg Sleep Quality Index; TST, total sleep time; SE, sleep efficiency; WASO, wake after sleep onset; ACSI-28, Athletic Coping Skills Inventory 28.

## Results

### Mindfulness

**Figure 2:**
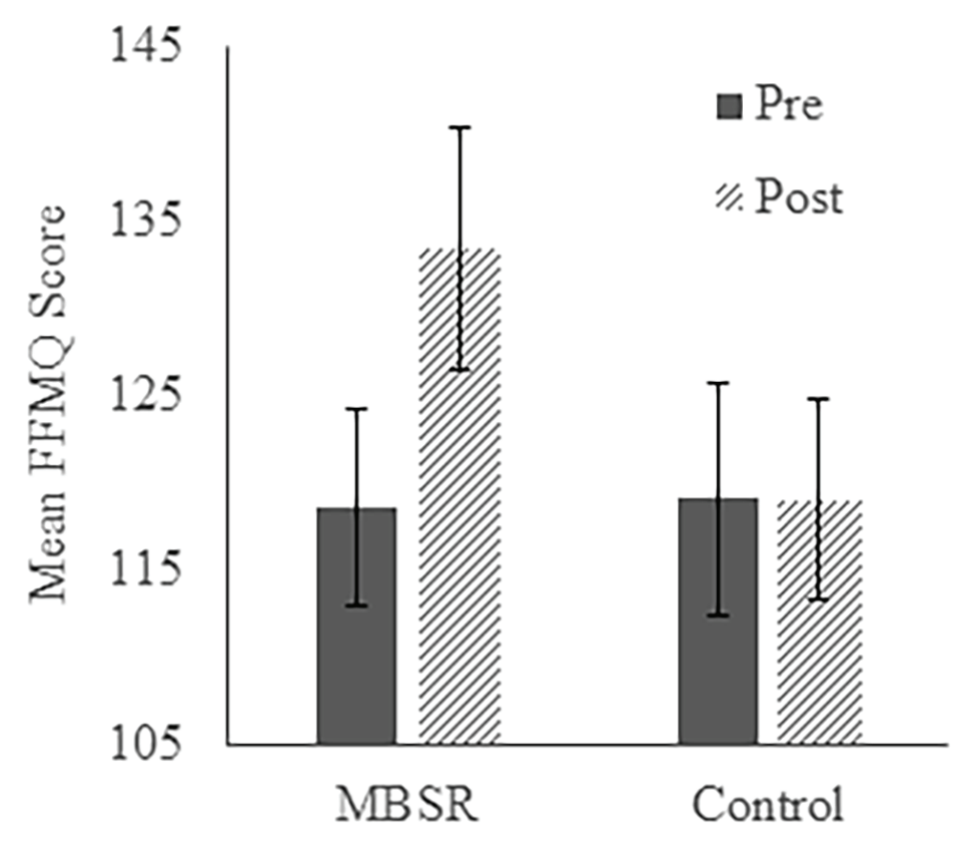
Average Five Facet Mindfulness Questionnaire (FFMQ) scores in the MBSR and Control groups at the pre-test and post-test time points. Higher scores reflect greater mindfulness. Error bars represent standard errors of means.

Independent samples *t*-tests showed no baseline difference between the MBSR and the Control groups on the Five Facet Mindfulness Questionnaire (FFMQ; see Table 1). Paired-samples *t*-tests indicated that scores significantly increased in the MBSR group [*t* = −2.646, *p* = 0.019, Cohen’s *d* = 0.683, 95% CI (−26.797, −2.803)] but not in the Control group [*t* = 0.000, *p* = 1.000, Cohen’s *d* = 0, 95% CI (−11.827, 11.827); Figure 2]. These results suggest that the MBSR course was effective at increasing mindfulness.

### Psychological Well-Being

**Figure 3:**
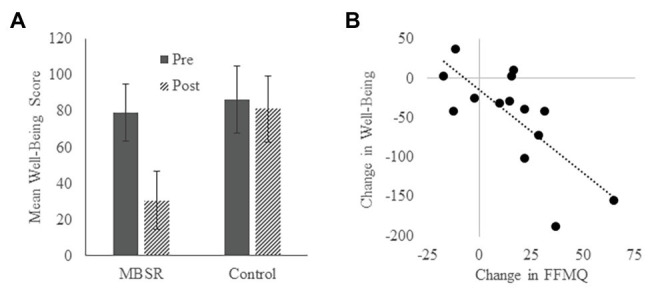
Psychological well-being. **(A)** Average psychological well-being scores in the MBSR and Control groups at the pre-test and post-test time points. Lower scores reflect greater well-being. Error bars represent standard errors of means. **(B)** Relationship between change in FFMQ score and change in psychological well-being score in the MBSR group.

There were no baseline differences between the MBSR group and the Control group on measures of psychological well-being (see Table 1). Composite scores of psychological well-being significantly declined (signifying improved well-being) in the MBSR group [*t* = 2.864, *p* = 0.013, Cohen’s *d* = 0.765, 95% CI (11.847, 84.616)] but not in the Control group [*t* = 0.377, *p* = 0.714, Cohen’s *d* = 0.114, 95% CI (−24.887, 35.037); Figure 3A]. Change in the well-being score was significantly negatively correlated with change in the FFMQ score in the MBSR group (*r* = −0.738, *p* = 0.003; Figure 3B). These results suggest that the MBSR course led to improved psychological well-being, and this improvement was associated with increased mindfulness.

### Sleep

**Figure 4:**
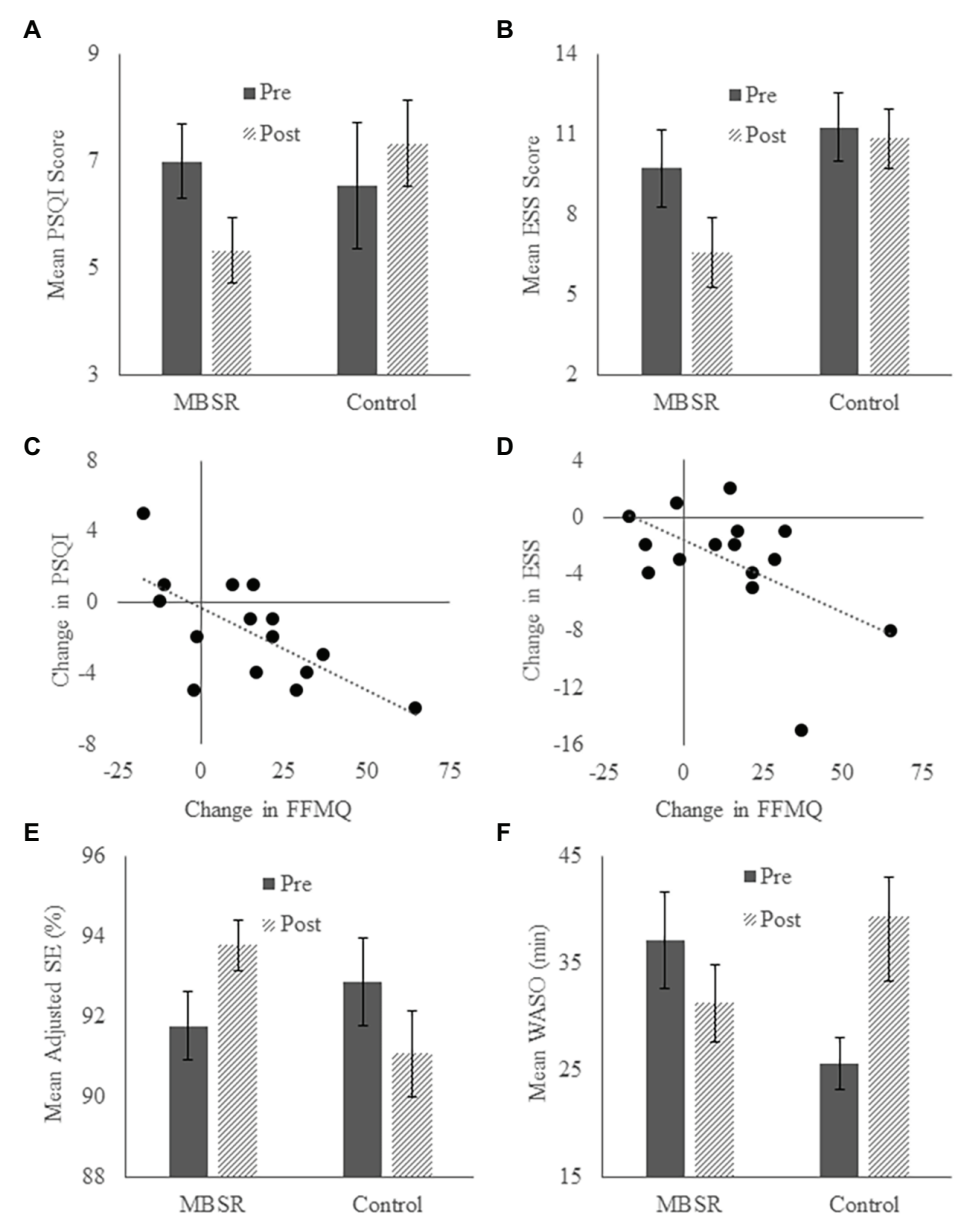
Sleep. **(A,B)** Average Pittsburg Sleep Quality Index (PSQI) and Epworth Sleepiness Scale (ESS) scores in MBSR and Control groups at the pre-test and post-test time points. Lower scores reflect better sleep quality and lower daytime sleepiness, respectively. **(C,D)** Relationships between change in FFMQ score and changes in PSQI and ESS scores. **(E,F)** Average percent sleep efficiency (SE) and minutes of wake after sleep onset (WASO) in MBSR and Control groups at the pre-test and post-test time points, as measured by actigraphy. Error bars represent standard errors of means.

There were no baseline differences between groups on subjective or actigraphy-derived sleep parameters (see Table 1). Pittsburg Sleep Quality Index (PSQI) scores non-significantly decreased (improved) in the MBSR group [*t* = 2.175, *p* = 0.047 (did not survive Bonferroni correction), Cohen’s *d* = 0.562, 95% CI (0.023, 3.310)] and non-significantly increased in the Control group [*t* = −2.277, *p* = 0.046 (did not survive Bonferroni correction), Cohen’s *d* = 0.687, 95% CI (−2.338, −0.025); Figure 4A]. Given the opposing directions of change in the two groups, we tested whether these patterns were significantly different using a 2 × 2 repeated-measures ANOVA with Time (Pre, Post) as a within-subjects variable and Group as a between-subjects variable. There was a significant Time × Group interaction [*F*(1,24) = 8.079, *p* = 0.009, partial *η*^2^ = 0.252], suggesting that MBSR benefited subjective sleep quality compared to the change observed in the Control group. The main effects of Time and Group were not significant (*p*’s > 0.35). Epworth Sleepiness Scale (ESS) scores significantly decreased in the MBSR group [*t* = 2.957, *p* = 0.010, Cohen’s *d* = 0.763, 95% CI (0.861, 5.406)], signifying reduced daytime sleepiness, and did not change in the Control group [*t* = 0.100, *p* = 0.922, Cohen’s *d* = 0.030, 95% CI (−1.935, 2.116); Figure 4B]. Change in FFMQ score was negatively correlated with change in PSQI score (*r* = −0.679, *p* = 0.005; Figure 4C) and change in ESS score (*r* = −0.535, *p* = 0.040; Figure 4D) in the MBSR group. These results suggest that the MBSR course led to improved subjective sleep quality and decreased subjective daytime sleepiness, and these improvements were associated with increased mindfulness.

With regard to actigraphy measures, TST increased non-significantly in both groups (*p*’s > 0.06). SE increased significantly in the MBSR group [*t* = −2.787, *p* = 0.024, Cohen’s *d* = 0.929, 95% CI (−3.155, −0.298)] and decreased non-significantly in the Control group [*t* = 0.967, *p* = 0.371, Cohen’s *d* = 0.365, 95% CI (−1.309, 3.019); Figure 4E]. WASO decreased non-significantly in the MBSR group [*t* = 2.025, *p* = 0.077, Cohen’s *d* = 0.675, 95% CI (−0.901, 13.878)] and increased non-significantly in the Control group [*t* = −1.398, *p* = 0.212, Cohen’s *d* = 0.528, 95% CI (−20.374, 5.557); Figure 4F]. Consistent with effects on subjective sleep measures, these results suggest that the MBSR course led to improved sleep quality. However, unlike subjective sleep measures, improvements in SE, and WASO were not correlated with change in FFMQ score (*p*’s > 0.27).

### Athletic Coping and Performance

**Figure 5:**
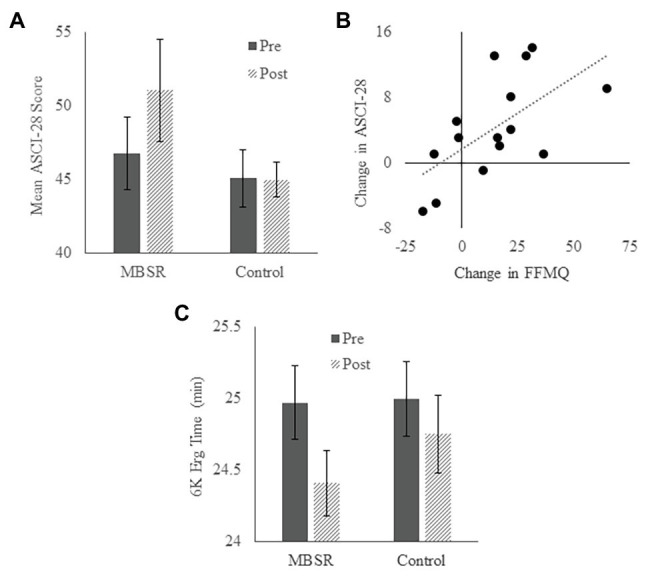
Athletic coping skills and rowing performance. **(A)** Average Athletic Coping Skills Inventory 28 (ACSI-28) scores in MBSR and Control groups at the pre-test and post-test time points. Higher scores reflect better coping skills. **(B)** Relationship between change in FFMQ score and change in ACSI-28 score. **(C)** Average time taken to complete the 6 K ergometer test in the MBSR and Control groups at the pre-test and post-test time points. Error bars represent standard errors of means.

There was no difference between groups on the Athletic Coping Skills Inventory 28 (ACSI-28) or the ergometer test at baseline (see Table 1). There was a significant increase in ACSI-28 scores in the MBSR group [*t* = −2.678, *p* = 0.018, Cohen’s *d* = 0.69, 95% CI (−7.683, −0.850)] and not the Control group [*t* = 0.302, *p* = 0.769, Cohen’s *d* = 0.091, 95% CI (−3.478, 4.569); Figure 5A]. Change in ACSI-28 score was positively correlated with change in FFMQ score in the MBSR group (*r* = 0.621, *p* = 0.013; Figure 5B). These results suggest that the MBSR course led to improved athletic coping skills, which was associated with increased mindfulness.

With regard to the ergometer test, there was a significant reduction in the time to completion in the MBSR group [*t* = 4.008, *p* = 0.002, Cohen’s *d* = 1.112, 95% CI (15.248, 51.567)] but not in the Control group [*t* = 1.117, *p* = 0.29, Cohen’s *d* = 0.337, 95% CI (−14.589, 43.912); Figure 5C]. Change in completion time was not correlated with change in FFMQ score in the MBSR group (*p* > 0.54), nor was it correlated with change in psychological well-being, athletic coping skills, or any sleep parameter (all *p*’s > 0.19). These results suggest that MBSR benefits objective rowing performance. However, improved physical performance was not associated with increased mindfulness in the current sample.

### Mediating Influence of Sleep

As previously described, improvement in mindfulness predicted improved sleep quality (PSQI score), improved psychological well-being, and improved athletic coping skills. Given the importance of sleep for psychological well-being and coping skills, we questioned whether improvement in sleep quality mediated the relationships between improved mindfulness and these other outcome variables. Mediation was tested using the causal steps method (MacKinnon et al., 2007).

To first determine whether mediation was plausible, we conducted simple linear regressions to assess the relationships between improved sleep quality and improved psychological well-being and coping skills. There was some evidence that change in PSQI score predicted change in psychological well-being score (*β* = 0.49, *p* = 0.075), and change in PSQI score did predict change in ACSI-28 score (*β* = −0.672, *p* = 0.006), suggesting that mediation was plausible in both cases. We next conducted multiple linear regressions to determine whether these relationships were still apparent when controlling for change in FFMQ score. Change in PSQI score predicted neither change in psychological well-being score (*β* = −0.052, *p* = 0.86) nor change in ACSI-28 score (*β* = −0.464, *p* = 0.12) when change in FFMQ score was included as a predictor variable. Thus, change in sleep quality did not appear to mediate the influence of increased mindfulness on psychological well-being or athletic coping skills in this study.

## Discussion

Here, we found that an 8-week MBSR course benefitted psychological well-being, sleep quality, athletic coping skills, and athletic performance in female collegiate rowers. Several of these benefits were correlated with increases in mindfulness, as measured by the FFMQ. While more research is needed to confirm these findings, results of this study suggest that the addition of mindfulness practice to athletic training programs could improve the quality of life and performance of student athletes.

We observed evidence that MBSR benefitted psychological well-being. Those assigned to the MBSR intervention showed significant improvement in psychological well-being, whereas those in the control group showed no improvement. Improvement in well-being in MBSR participants was related to increased mindfulness. This finding is consistent with a large body of evidence indicating that mindfulness-based interventions improve well-being in patient populations as well as non-clinical populations, including college students (Grossman et al., 2004; Chiesa and Serretti, 2009; McConville et al., 2017).

We also observed that MBSR benefitted both subjective and objective sleep quality. With regard to subjective sleep measures, MBSR participants reported improved sleep quality and significantly decreased daytime sleepiness, whereas control participants reported worsened sleep quality and no change in sleepiness. These improvements in the MBSR group were associated with increased mindfulness. The pattern of results in subjective sleep quality was reinforced by actigraphy outcomes, as we observed significant improvement in SE and decreased WASO in MBSR participants and somewhat reduced SE and higher WASO in control participants. Interestingly, this was the case even though actigraphy was measured a few weeks after completion of the MBSR course, perhaps suggesting sustained effects on sleep quality in the short-term.

These results are consistent with many past findings indicating that mindfulness-based interventions improve subjective sleep quality (Rusch et al., 2019). While some evidence suggests mindfulness training can improve objective sleep parameters in populations with sleep problems such as insomniacs and cancer patients and survivors (Gross et al., 2011; Garland et al., 2014; Lengacher et al., 2015), effects have rarely been observed in non-clinical populations. Based on evidence that meditation practice before sleep reduced arousal and improved subjective sleep quality in athletes (Li et al., 2018), MBSR participants in the current study were instructed to practice daily before sleep. This practice before sleep may have contributed to benefits in sleep quality. It must be noted, however, that we did not measure participant compliance with this instruction.

Finally, we observed evidence that MBSR benefitted athletic coping skills and rowing performance. After 8 weeks, MBSR participants showed significantly improved coping skills whereas control participants showed no change. Improvement in coping skills was related to increased mindfulness. After 6 weeks, MBSR participants showed significantly reduced time required to complete the 6K ergometer test, whereas control participants did not show significant improvement. These findings are consistent with previous studies showing a positive effect of trait mindfulness and mindfulness interventions on athletic coping skills and subjective performance (Pineau et al., 2014; Röthlin et al., 2016; Josefsson et al., 2017; Vidic et al., 2017). While some evidence suggests that mindfulness training can improve performance in precision sports such as shooting and dart throwing (Solberg et al., 1996; John et al., 2011; Zhang et al., 2016), few controlled experimental studies have investigated effects in non-precision sports (Bühlmayer et al., 2017). The current results suggest that mindfulness training may indeed benefit non-precision sports such as rowing. However, more randomized controlled studies are needed to examine this matter.

Since psychological well-being, sleep quality, and athletic coping skills are all important for peak athletic performance, it seems likely that improvements in these factors contributed to improvement in physical performance, though we did not see evidence of such relationships in the current study. However, it is also possible that increased mindfulness directly benefits athletic performance *via* attentional strategy used during performance. An associative strategy is characterized by focus on performance-relevant cues including sensory feedback such as muscle tension, respiration, and heart rate, and has been linked to better performance in endurance sports including rowing (Scott et al., 1999; Connolly and Janelle, 2003). Since mindfulness training promotes moment-to-moment awareness of such internal cues, it stands to reason that such training may promote the use of an associative attention strategy which could directly benefit physical performance. However, associative strategies can be more or less beneficial depending on the locus of attention (Comani et al., 2014; Bertollo et al., 2015; di Fronso et al., 2018). Future studies could investigate effects of mindfulness interventions on attention strategy employed during performance.

Limitations of this study should be considered. Notably, the control group did not undergo any active control or sham intervention, raising the question of whether the effects of MBSR could have been due to non-specific aspects of the intervention (e.g., weekly group meeting and discussion). While non-specific effects cannot be ruled out, the relationships with improved mindfulness strongly suggest that the benefits were associated with the specific content of the course. Another point is that group assignment was not entirely random but based on availability during the MBSR class time for some individuals. Since the groups were well-matched with regard to baseline performance, it seems unlikely that this feature of group assignment would have influenced the results. Nonetheless, future studies using complete randomization will be valuable. An additional consideration is that the efficacy of the PSQI as a clinical assessment tool in athletes has been called into question (Samuels et al., 2016). Notably, this is not problematic in our study design, as we used the PSQI to assess within-subjects change in sleep quality rather than for diagnostic purposes. Finally, this study measured only one index of objective athletic performance, completion time on the ergometer test, and at only two time points. This design was based on the natural training schedule of the team. However, future studies could incorporate multiple indices of performance assessed at multiple time points during the intervention and afterward in order to thoroughly determine the effects of MBSR on athletic performance and the time course of any such effects. Overall, while more research is needed, results of this study suggest that incorporating mindfulness training into athletic programs could improve psychological well-being, sleep quality, and performance of student athletes.

## Data Availability Statement

The raw data supporting the conclusions of this article will be made available by the authors, without undue reservation.

## Ethics Statement

The studies involving human participants were reviewed and approved by University of Massachusetts Amherst Institutional Review Board. The patients/participants provided their written informed consent to participate in this study.

## Author Contributions

BJ and SK designed the experiment, analyzed data, and wrote the manuscript. SK collected data. MM conducted the mindfulness-based stress reduction course and aided in aspects of experimental design. RS supervised the entire project. All authors reviewed and gave feedback on the manuscript. All authors contributed to the article and approved the submitted version.

### Conflict of Interest

MM was employed by Amherst Mindfulness.

The remaining authors declare that the research was conducted in the absence of any commercial or financial relationships that could be construed as a potential conflict of interest.
